# Reversal of direct oral anticoagulants in hemophilia treatment: ASH meeting 2015

**DOI:** 10.1007/s12254-016-0284-2

**Published:** 2016-09-12

**Authors:** Clemens Feistritzer, Stefan Schmidt

**Affiliations:** Internal Medicine V – Hematology and Oncology, Medical University of Innsbruck, Anichstraße 35, 6020 Innsbruck, Austria

**Keywords:** Thrombophilia screening, Pregnancy, Anticoagulation, Antidote, Hemophilia A, Inhibitor

## Abstract

During the 57^th^ annual meeting of the American Society of Hematology 2015 in Orlando, Florida, various aspects in the field of hemostaseology were presented. The Choosing Wisely® campaign pointed out the importance of the critical use of diagnostic tools to rule out pulmonary embolism and questioned the relevance of thrombophilia testing in women undergoing routine infertility evaluation. Furthermore, the approval of idarucizumab, a specific antidote for the reversal of the anticoagulant effects of the direct thrombin inhibitor dabigatran, was highlighted. Finally, hemophilia specialists awaited the results of the SIPPET Trial, which were presented for the first time during the plenary session. Replacement therapy of previously untreated hemophilia A patients with plasma-derived factor VIII containing von Willebrand factor resulted in a lower incidence of inhibitors compared with patients treated with recombinant factor VIII.

## Take-home messages:

- Evidence-based use of diagnostic approaches (e. g., CT scans, thrombophilia testing) must be demanded in hemostaseology.
- Specific antidotes, e.g., idarucizumab for dabigatran, are needed for the reversal of the anticoagulant effects of direct oral anticoagulants.
- In hemophilia A patients, replacement therapy with plasma-derived factor VIII containing von Willebrand factor led to a lower incidence of inhibitors compared with treatment with recombinant factor VIII.

## Introduction

The annual meeting of the American Society of Hematology (ASH) 2015 was held in Orlando, Florida. As expected, the main focus of the meeting was on novel developments in hematologic diseases; however, different topics in the field of hemostaseology – from thrombophilia testing to treatment options in hemophilia – were also discussed. It should be pointed out that very important issues in hemostaseology, including perioperative bridging of anticoagulation in patients with atrial fibrillation [1] or reversal of direct oral anticoagulants [2, 3], were already presented during the meeting of the International Society of Thrombosis and Haemostasis in Toronto in June and therefore published earlier this year. The highlight of the congress for hemophilia specialists was undoubtedly the first presentation of the results of the SIPPET trial, which will probably have a huge impact on the choice of factor VIII product (plasma-derived vs. recombinant) for the treatment of patients with hemophilia A [4].

This short review summarizes the clinically most relevant topics in hemostaseology from the ASH meeting 2015 in the context of previously published data.

## The ASH Choosing Wisely® campaign 2015

Initiated in 2013, the ASH Choosing Wisely® Task Force annually announces hematologic tests and treatments of questionable clinical value, which increase costs with a low likelihood of benefit and even expose patients to potential harm. Noteworthy, in the last 2 years several of the tests and treatments highlighted were in the field of coagulation, reflecting the need for evidence-based treatment guidelines [5, 6]. This year the ASH committee released a list of tests and procedures based on Non-ASH Choosing Wisely® recommendations from other medical societies with relevance to hematology. Nevertheless, also in 2015, two out of five recommendations were related to hemostaseology. One of them was initially from 2012 from the American College of Radiology and recommended against the imaging for suspected pulmonary embolism (PE) without moderate or high pretest probability of PE. The probability of PE can be assessed by using clinical symptoms and the patient history in the Wells score [7, 8] (see Table 1). In the case of a low probability, PE might be ruled out by normal d-dimer levels without further imaging and thereby unnecessary exposure to radiation can be avoided. The specificity of this approach has been prospectively shown in several publications [9]. Another recommendation was based on the American Society for Reproductive Medicine. The authors questioned the clinical relevance of routinely ordering thrombophilia testing for patients undergoing a routine infertility evaluation. In several studies an association between different types of thrombophilia and miscarriage, pregnancy loss, as wells as pregnancy complications was shown; however, the numbers are within a very wide range and inconsistent (summarized in [10]). Moreover, large prospective studies showing that the use of either low-molecular-weight heparin or platelet inhibition as well as the combination of both can lead to a higher chance of a successful pregnancy in women with thrombophilia are lacking [11]. In conclusion, thrombophilia testing does not affect the treatment of infertile woman.

**Table 1:** Clinical assessment of the probability of PE using Wells’ Criteria [6, 7]

*Symptoms and patient history*	*Points*
Clinical symptoms of DVT (leg swelling, pain with palpation)	3.0
Other diagnosis less likely than PE	3.0
Heart rate >100/min	1.5
Immobilization (>3 days) or surgery in the previous 4 weeks	1.5
Previous DVT/PE	1.5
Hemoptysis	1.0
Malignancy	1.0
*Traditional clinical probability assessment (Wells’ criteria)*	*Score*
High	>6.0
Moderate	2.0–6.0
Low	<2.0

*DVT* deep vein thrombosis, *PE* pulmonary embolism

## Idarucizumab: antidote for the reversal ofanticoagulant effects of dabigatran

**Fig. 1:**
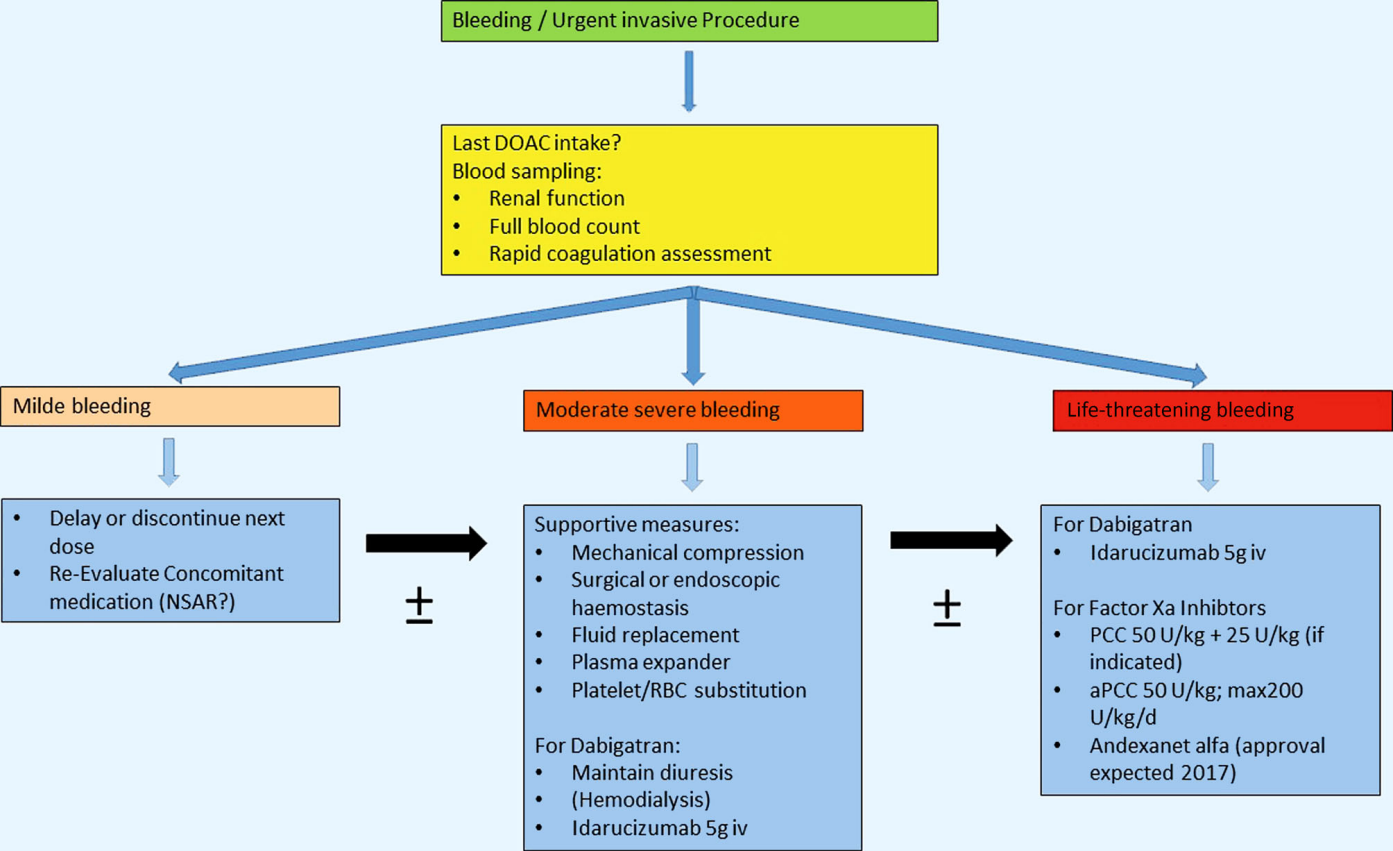
Recommendations for the treatment of bleeding complications or urgent interventions occurring during treatment with direct oral anticoagulants (adapted from Heidbuchel et al. [20])

In general, all clinical studies of patients suffering from atrial fibrillation or deep vein thrombosis/pulmonary embolism have shown positive risk–benefit analysis of direct oral anticoagulants (DOAC) compared with vitamin K antagonists (VKA), particularly when it comes to bleeding complications with the exception of gastrointestinal bleeds. Especially the risk of intracerebral bleeding was lower in patients treated with DOACs than with VKA [12–17]. Lately, these findings have been confirmed by real-life data [18, 19]. However, bleeding complications and other emergency situations like accidents or the need for urgent surgery can obviously still occur, requiring rapid reversal of these drugs. Until 2015 the treatment options in emergency situations were limited. Guidelines recommended the use of supportive measures (e. g., mechanical compression, surgical procedures, fluid replacement) in the case of moderate to severe bleedings; in life-threatening bleedings, prothrombin complex concentrates (PCC) or activated PCC (factor VIII inhibitor bypass activity, FEIBA) should be administered [20]. Alternatively to PCC, the thrombin inhibitor dabigatran can be removed by hemodialysis ([21] Fig. 1). The recommendation for the use of PCC is primarily based on in vitro assays, ex vivo tests, or animal studies. However, these studies revealed variable responses of the DOACs toward the PCC [22, 23], but in the case of an emergency an exact prediction of the potential effects is necessary. Therefore for the reversal of DOACs, specific antidotes with a rapid onset of action, high binding affinity to the target protein, and no effects on the coagulation cascade itself are needed. During the ASH meeting in 2015, Kenneth Bauer highlighted Idarucizumab (Praxbind®), the first specific antidote for the thrombin inhibitor dabigatran in the session on clinical applications of newly approved drugs. Idarucizumab is a humanized monoclonal antibody fragment that has been specifically developed for the reversal of dabigatran [24]. Earlier studies have demonstrated that idarucizumab restores dabigatran-prolonged coagulation parameters to baseline values, not only in healthy volunteers, but also in the elderly and patients with chronic kidney disease [25]. Idarucizumab binds specifically to unbound and thrombin-bound dabigatran with an affinity approximately 350-fold higher than the affinity of dabigatran for thrombin. Once dabigatran is complexed to idarucizumab, the anticoagulant effects are neutralized. In a recently published phase III trial, idarucizumab was used in a fixed dose of 2 × 2.5 g in patients with either life-threatening bleeding or requiring emergency surgery or invasive procedures. Idarucizumab not only completely reversed the anticoagulant effects of dabigatran within minutes as demonstrated by various laboratory tests, but it also led to a normal intraoperative hemostasis in the majority of patients based on the assessment of the surgeons involved during the procedures [2]. Finally, in late 2015, idarucizumab was approved by the Food and Drug Administration (FDA) and European Medicines Agency (EMA) for the treatment of dabigatran-induced bleedings.

A different approach is used for the specific reversal of the factor Xa inhibitors. Andexanet alfa (Annexa®) is a recombinant modified human factor Xa decoy protein that is catalytically inactive but retains the ability to bind factor Xa inhibitors in the active site, thereby restoring the activity of endogenous factor Xa [26]. In phase II/III studies, intravenous administration of andexanet alfa resulted in dose-dependent rapid reversal of the anticoagulant effects of the factor Xa inhibitors in healthy volunteers. Reversal was assessed as the reduction in anti-factor Xa activity and unbound factor Xa inhibitor concentrations, as well as the restoration of thrombin generation. Because of the short half-life of andexanet alfa, this agent was also administered as a bolus injection followed by continuous infusion [3, 25]. A study focusing on the effects of andexanet alfa on bleeding complications during treatment with factor Xa inhibitors is still ongoing. Owing to the urgent need for a specific antidote for factor Xa inhibitors, the approval of the drug is expected as soon as the results of the ongoing study are available.

## Treatment–related development of anti-factor VIII antibodies

Finally, Flora Peyvandi presented the data of the SIPPET study (Survey of Inhibitors in Plasma-Product Exposed Toddlers) during the plenary session of the 2015 ASH meeting [4]. The aim of this prospective, multicenter, open-label, randomized study was to compare the incidence of factor VIII (FVIII) inhibitors with plasma-derived FVIII concentrates containing von Willebrand factor versus recombinant FVIII concentrates in previously untreated patients (PUPs) with severe hemophilia. Inhibitor development is the most challenging complication of hemophilia treatment. It not only causes a high economic burden for a chronic disease, but also leaves FVIII treatment ineffective thereby increasing the risk of bleeding complications.

A number of risk factors have been identified for the development of FVIII inhibitors in children with hemophilia A, including: disease severity, defects in the FVIII gene, ethnicity, and number of exposure days to FVIII [27, 28]. There is also an ongoing discussion whether recombinant FVIII products are more immunogenic than plasma-derived products. In 2013, the RODIN (Research of Determinants of INhibitor development) study, to date the largest multicenter observational cohort study included 574 PUPs with severe hemophilia A, published its findings. In this study, 177 patients (32 %) developed inhibitors; however, recombinant and plasma-derived FVIII products conferred similar risks of inhibitor development. The content of von Willebrand factor in the products and switching among products were not associated with the risk of inhibitor development. Interestingly, second-generation full-length recombinant products were associated with an increased risk, as compared with third-generation products [29]. However, the study was criticized because of the partially retrospective design as well as the unbalanced cohort groups. In the SIPPET trial, 303 patients were screened, and finally 251 were analyzed. In general, the higher number of PUPs developing inhibitors compared with those in the RODIN trial was striking. Inhibitors developed in 26.8 % of patients treated with plasma-derived factor VIII and 44.5 % with recombinant factor VIII; recombinant factor VIII was associated with an 87 % higher incidence than plasma-derived factor VIII. When the analysis was restricted to recombinant factor VIII products other than second-generation full-length recombinant factor VIII, the effect estimates remained similar for all inhibitors. In contrast to the data of the RODIN trial, Peyvandi and her team concluded that PUPs treated with plasma-derived FVIII containing von Willebrand factor had a lower incidence of inhibitors than those treated with recombinant FVIII.

## Abbreviations

ASH: American Society of Hematology
DOAC: direct oral anticoagulants
FVIII: factor VIII
PE: pulmonary embolism
PUP: previously untreated patients
VKA: vitamin K antagonists
